# Immunogenicity against wild-type and Omicron SARS-CoV-2 after a third dose of inactivated COVID-19 vaccine in healthy adolescents

**DOI:** 10.3389/fimmu.2023.1106837

**Published:** 2023-03-06

**Authors:** Daniel Leung, Carolyn A. Cohen, Xiaofeng Mu, Jaime S. Rosa Duque, Samuel M. S. Cheng, Xiwei Wang, Manni Wang, Wenyue Zhang, Yanmei Zhang, Issan Y. S. Tam, Jennifer H. Y. Lam, Sau Man Chan, Sara Chaothai, Kelvin K. H. Kwan, Karl C. K. Chan, John K. C. Li, Leo L. H. Luk, Leo C. H. Tsang, Nym Coco Chu, Wilfred H. S. Wong, Masashi Mori, Wing Hang Leung, Sophie Valkenburg, Malik Peiris, Wenwei Tu, Yu Lung Lau

**Affiliations:** ^1^ Department of Paediatrics and Adolescent Medicine, The University of Hong Kong, Hong Kong, Hong Kong SAR, China; ^2^ School of Public Health, The University of Hong Kong, Hong Kong, Hong Kong SAR, China; ^3^ HKU-Pasteur Research Pole, School of Public Health, The University of Hong Kong, Hong Kong, Hong Kong SAR, China; ^4^ Research Institute for Bioresources and Biotechnology, Ishikawa Prefectural University, Nonoichi, Japan; ^5^ Department of Microbiology and Immunology, Peter Doherty Institute for Infection and Immunity, University of Melbourne, Melbourne, VIC, Australia; ^6^ Centre for Immunology & Infection C2i, Hong Kong, Hong Kong SAR, China

**Keywords:** COVID-19, vaccine, CoronaVac, Omicron, adolescent

## Abstract

**Introduction:**

Two doses of inactivated SARS-CoV-2 vaccine CoronaVac cannot elicit high efficacy against symptomatic COVID-19, especially against the Omicron variant, but that can be improved by a third dose in adults. The use of a third dose of CoronaVac in adolescents may be supported by immunobridging studies in the absence of efficacy data.

**Methods:**

With an immunobridging design, our study (NCT04800133) tested the non-inferiority of the binding and neutralizing antibodies and T cell responses induced by a third dose of CoronaVac in healthy adolescents (N=94, median age 14.2 years, 56% male) compared to adults (N=153, median age 48.1 years, 44% male). Responses against wild-type (WT) and BA.1 SARS-CoV-2 were compared in adolescents. Safety and reactogenicity were also monitored.

**Results:**

A homologous third dose of CoronaVac further enhanced antibody response in adolescents compared to just 2 doses. Adolescents mounted non-inferior antibody and T cell responses compared to adults. Although S IgG and neutralizing antibody responses to BA.1 were lower than to WT, they remained detectable in 96% and 86% of adolescents. T cell responses to peptide pools spanning only the mutations of BA.1 S, N and M in adolescents were preserved, increased, and halved compared to WT respectively. No safety concerns were identified.

**Discussion:**

The primary vaccination series of inactivated SARS-CoV-2 vaccines for adolescents should include 3 doses for improved humoral immunogenicity.

## Highlights

1. A third dose of CoronaVac is needed for improved immunogenicity in healthy adolescents
2. Non-inferiority of antibody and T cell responses in adolescents versus adults
3. BA.1 S IgG and neutralizing antibodies were detectable in 96% and 86% adolescents after dose 3
4. T cell responses against BA.1 mutations in S, N and M were preserved, increased, and halved, respectively

## Introduction

Inactivated vaccines against COVID-19 such as CoronaVac are widely used with more than 4 billion doses distributed worldwide because of simpler manufacturing requirements and greater vaccine stability during transport (1). Real-world vaccine effectiveness studies have also shown that 2 doses of inactivated vaccines could strongly protect against severe COVID-19 but less so against mild disease (2, 3). In comparison to the mRNA COVID-19 vaccines widely in use, 2 doses of inactivated COVID-19 vaccines elicit weaker neutralization responses yet higher T cell responses in adults (4), as well as in adolescents as we have shown (5). There is a growing consensus that the primary series of inactivated COVID-19 vaccines should include 3 doses, similar to other routinely used inactivated vaccines such as the inactivated polio vaccine. Homologous third dose of CoronaVac has been shown to improve vaccine effectiveness against mild and severe COVID-19 in adults (3, 6). However, as of September 2022, there is currently no published data on the paediatric use of 3 doses of CoronaVac.

Vaccine effectiveness, especially against mild disease, is susceptible to waning over time as well as to antigenically divergent variants of concern (3, 7). Neutralizing antibody escape by the newly emergent Omicron variant may account for high transmission in populations with high vaccine coverage (8, 9). On the other hand, T cell responses in adults are mostly (~80%) preserved against the Omicron variant as most immunodominant T cell epitopes are unaffected (10–12), which may explain the preservation of vaccine effectiveness against severe outcomes with Omicron variant (3). Data from the United Kingdom showed that in contrast to adults, adolescents are not at significantly lower risk of hospitalization due to Omicron relative to Delta (13), and paediatric COVID-19-associated hospitalizations increased rapidly during the Omicron outbreak in South Africa (14). In adults, a third dose of COVID-19 vaccine boosted neutralizing antibody and T cell response against the Omicron variant (11, 15), yet this remains unknown in adolescents.

To inform the paediatric use of CoronaVac, an inactivated COVID-19 vaccine, amid the spread of Omicron, we sought to determine the safety and immunogenicity of a third dose of CoronaVac in healthy adolescents. We adopted an immunobridging design, where adolescents were tested for whether various immunogenicity outcomes, including antibody binding and avidity, neutralizing and non-neutralizing antibody functions, and T cell responses against the wild-type (WT) virus (5), were non-inferior to those in adults. The goal is to support the extension of age group indication for the third dose of CoronaVac in the absence of efficacy data in adolescents, based on the established effectiveness of a homologous third dose of CoronaVac in adults (3, 6). In addition, immunogenicity against Omicron BA.1 was also assessed.

## Methods

### Study design

COVID-19 Vaccination in Adolescents and Children (COVAC; NCT04800133) is a non-randomized immunobridging study of BNT162b2 and CoronaVac in adolescents and children, as previously described (5, 16). The University of Hong Kong (HKU)/Hong Kong West Cluster Hospital Authority Institutional Review Board (UW21-157) authorized this study. ClinicalTrials.gov

### Participants

The current analysis included adolescents aged 11-17 years and adults ≥18 years at the time of dose 1 who received 3 intramuscular doses of CoronaVac. The exclusion were history of COVID-19, severe allergy, major neuropsychiatric issues, immune compromise conditions, blood transfusion within 60 days, significant bleeding tendency, and pregnancy or breastfeeding.

### Procedures

Participants were recruited in Hong Kong from schools, media, or referral. Written informed consent was obtained from participants aged ≥18 years or above. Informed assent was obtained from underage participants and written consent was obtained from their parents or legally acceptable representatives. Vaccination consisted of three doses of 0.5 mL inactivated virus vaccine that contains 600SU of SARS-CoV-2 CZ02 strain whole virus antigen. Doses 1 and 2 were administered 28-35 days apart, while dose 3 was given ≥84 days after dose 1. The vaccination interval was chosen after the finding of limited durability of the 2-dose antibody response during an evolving pandemic and likely benefits of more persistent prime-boost interval (17). Blood was sampled on the day of dose 3 and 13-42 days following dose 3 to detect the expected peak antibody response and short-term cellular response after dose 3 (18).

#### Safety data collection

Participants were observed for 15 minutes after each vaccine injection. Prespecified adverse reactions (ARs) were recorded in an online or paper-based diary for 7 days after vaccine administration. Unsolicited adverse events were captured for 28 days after each vaccine dose. There will be ongoing surveillance for severe adverse events include hospitalizations, life-threatening complications, disabilities, deaths, birth defects in offspring, and breakthrough COVID-19 for 3 years. The study investigators determined whether there was causal relationship of the study vaccine with the reported adverse effects.

#### S-RBD IgG, N IgG and N-CTD IgG, surrogate virus neutralization test (sVNT) and plaque reduction neutralization test (PRNT)

Clotted blood and the serum from the participants was maintained at -80° C. Sera were inactivated at 56° C for 30 minutes before performance of the SARS-CoV-2 S receptor-binding domain (S-RBD) IgG, N and N-CTD IgG enzyme-linked immunosorbent assay (ELISA), sVNT (GenScript Inc, Piscataway, USA) and PRNT as previously described and validated according to the manufacturer’s instructions (19–21). The cut-offs for ELISA-based tests were derived from mean of OD + 3SD of pre-pandemic samples. For sVNT, the cut-off was provided by the manufacturer. The cut-off for the PRNT was set at 1:10, which was the lowest dilution demonstrating inhibition to the virus.

In summary, S-RBD IgG ELISA plates were coated with 100 ng/well of purified recombinant S-RBD in PBS buffer overnight and 100 μL Chonblock Blocking/Sample Dilution (CBSD) ELISA buffer (Chondrex Inc, Redmond, USA) was added. This mixture remained at room temperature (RT) for 2 hours. Sera at 1:100 dilution in CBSD ELISA buffer were added to the wells at 37 for 2 hours. The wells were washed with PBS containing 0.1% Tween 20, followed by the addition of horseradish peroxidase (HRP)-conjugated goat anti-human IgG (1:5,000) (GE Healthcare, Chicago, USA) for 1 hour at 37°C. These were washed with PBS containing 0.1% Tween 20 for five times, and then 100 μL HRP substrate (Ncm TMB One, New Cell & Molecular Biotech Co. Ltd, China) was added and kept for 15 minutes. This reaction was ceased with 50 μL 2 M H_2_SO4. The OD of the mixture was analyzed in a Sunrise absorbance microplate reader (Tecan, Männedorf, Switzerland) at 450 nm wavelength. The background OD in the PBS-coated control wells with the sera was subtracted from each final OD reading. OD450 values below the cut-off of 0.5 were imputed as 0.25.

For N IgG and N-CTD IgG, the 96-well ELISA plates (Nunc MaxiSorp, Thermo Fisher Scientific) were coated with 125 ng (N) or 40.3 ng (N-CTD) purified recombinant protein in PBS buffer overnight. 100 μL Chonblock blocking/sample dilution ELISA buffer (Chondrex Inc, Redmon, US) was added to the plates, which were incubated for 1 hour at room temperature. Afterwards, the sera were diluted to 1:100 in Chonblock blocking/sample dilution ELISA buffer. Sera were added to the ELISA plates, which were incubated at 37 for 2 hours. Each well was washed with PBS containing 0.1% Tween 20 and incubated at 37 for 1 hour with anti-human IgG secondary antibody (1:2500, Thermo Fisher Scientific). The plates were washed five times with PBS containing 0.1% Tween 20, and 100 μL of HRP substrate (Ncm TMB One; New Cell and Molecular Biotech Co. Ltd, Suzhou, China) was added into each well. After a 15-minute incubation period, the reaction was stopped with 50 μL of 2M H_2_SO_4_ solution. OD450 was analyzed using an absorbance microplate reader.

10 μL of each sera was used for sVNT, with positive and negative controls prepared by dilution of 1:10 mixed with same volume of HRP-conjugated WT SARS-CoV-2 S-RBD (6 ng). The mixtures were incubated at 37 for 30 minutes, followed by the addition of 100 μL of sample to the microtitre plate wells coated with the recombinant angiotensin-converting enzyme-2 (ACE-2) receptor. The plates were sealed for 15 minutes at 37 and then washed with wash-solution and tapped dry. 100 μL of 3,3’,5,5’-tetramethylbenzidine (TMB) was then added, followed by incubation for 15 minutes at RT in the dark. 50 μL of Stop Solution was added. The absorbance was recorded at 450 nm. The % inhibition was calculated using the formula: (1-sample OD value/negative control OD value) x100%. Inhibition % below 30%, which was the limit of quantification (LOQ), was imputed as 15%.

PRNT duplicates were performed in a biosafety level 3 facility. Serial serum dilutions at 1:10 to 1:320 were incubated with ~30 plaque-forming units of SARS-CoV-2 BetaCoV/Hong Kong/VM20001061/2020 virus (WT) or hCoV-19/Hong Kong/VM21044713_WHP5047-S5/2021 (Omicron BA.1) for 1 hour at 37 in culture plates (Techno Plastic Products AG, Trasadingen, Switzerland) (8). We added the virus-sera mixtures onto Vero-E6 TMPRSS2 cell monolayers, which were then placed in a 5% CO2 incubator for 1 hour at 37°C. After overlaying with 1% agarose in cell culture medium, these plates were incubated for 3 days while fixed and stained. The antibody titres were defined as the reciprocal of the highest dilution of serum resulting in a >=90% (PRNT90) or >50% (PRNT50) reduction in the plaque numbers. Values above 1:320 were imputed as 1:640 and those below 10 were imputed as 5.

#### S IgG, avidity and FcγRIIIa-binding

We diluted the antigens for antibody detection in PBS and coated the plates (Nunc MaxiSorp, Thermofisher Scientific) with 250 ng/mL WT (AcroBiosystems) or Omicron BA.1 (AcroBiosystems) SARS-CoV-2 S protein for IgG and IgG avidity assessment and 500 ng/mL ancestral (Sinobiological) or Omicron BA.1 (AcroBiosystems) S for FcγRIIIa-binding detection. ORF8 protein of 300 ng/mL was coated at 37 for 2 hours. The plates were blocked with 1% FBS in PBS for 1 hour, followed by incubation with 1:100 HI sera diluted in 0.05% Tween-20/0.1% FBS in PBS for 2 hours for IgG detection, and 1:50 for 1 hour at 37 for FcγRIIIa-binding detection, prior to rinsing. For avidity, plates with 8M were washed with urea 3 times. IgG was measured after 2 hours of incubation period with anti-IgG-HRP (1:5000; G18-145, BD), HRP revealed with addition of stabilized hydrogen peroxide and tetramethylbenzidine (R&D systems) for 20 minutes. The reaction was terminated with 2N H2SO4, which was then analyzed at 450 nm wavelength with an absorbance microplate reader (Tecan Life Sciences). Similarly, FcγRIIIa-binding antibodies were assessed after incubation with biotinylated FcγRIIIa-V158 at 100 ng/mL for 1 hour at 37 after streptavidin-HRP (1:10000, Pierce).

#### T cell responses

Peripheral blood mononuclear cells (PBMCs) were extracted and maintained at -80° C. Thawed PBMCs were placed in 10% human AB serum supplemented RPMI medium for 2 hours. The PBMCs were stimulated with sterile ddH_2_O or 1 µg/mL overlapping peptide pools representing the WT SARS-CoV-2 S, N and M proteins (Miltenyi Biotec, Bergisch Gladbach, Germany), or BA.1 S mutation pool and WT S reference pool (Miltenyi Biotec, Bergisch Gladbach, Germany), Omicron BA.1 N mutation pool, WT N reference pool, BA.1 M mutation pool and WT M reference pool (peptide sequences in **Supplementary Table 6**; synthesized by ChinaPeptides Co., Ltd) in 1 µg/mL anti-CD28 and anti-CD49d costimulatory antibodies (clones CD28.2 and 9F10, Biolegend, San Diego, USA) for 16 hours, followed by the addition of 10 µg/mL brefeldin A (Sigma, Kawasaki, Japan) (22). The PBMCs were then washed and stained for CD3 (HIT3a, 1:60), CD4 (OKT4, 1:60), CD8 (HIT8a, 1:60), IFN-γ (B27, 1:15), IL-2 (MQ1-17H12, 1:15) (Biolegend, San Diego, USA) and fixable viability dye (eBioscience, Santa Clara, USA, 1:60). Flow cytometry was performed by the LSR II (BD Biosciences, Franklin Lakes, USA). Flowjo v10 software (BD, Ashland, USA) was used to analyze the data. Calculation of measured IFN-γ^+^ or IL-2^+^ T cells were performed by deducting the background (sterile ddH2O) data, which are presented as the percentages of CD4^+^ or CD8^+^ T cells (23). T cell responses against the peptide pool was considered positive if the cytokine-expressing cell frequency was ≥0.005% and the stimulation index was >2. Negative values were imputed as 0.0025%. The total T cell responses against S, N and M peptide pools were summed and the cut-off of 0.01% was used.

### Outcomes

For the current analysis, the primary immunogenicity outcomes were S-specific antibody markers, which included the S IgG and S-RBD IgG levels, sVNT %inhibition, 90% and 50% PRNT titres, S IgG avidity and FcγRIIIa-binding, and the total and separate S, N and M-specific IFN-γ^+^ and IL-2^+^ CD4^+^ and CD8^+^ T cell responses measured by flow cytometry 13-42 days after the third dose of CoronaVac. The primary reactogenicity outcomes were ARs and anti-pyretic use within 7 days after vaccine injection.

The secondary immunogenicity outcomes were N and N-CTD IgG levels, and antibody and T cell responses against Omicron BA.1. For safety, the secondary outcomes were AEs within 28 days post-vaccination and SAEs during the study period.

### Statistical analyses

#### Sample size and power estimation

G*Power (Heinrich-Heine-Universität Düsseldorf, Düsseldorf, Germany) and Sampsize (sampsize.sourceforge.net) were used for the power calcuation. For primary immunogenicity objectives, when comparing the peak geometric mean (GM) immunogenicity outcomes between adolescents and adults, 61 participants in each group would allow two-sided tests with α=0.05 and 99% power to detect a difference of 0.51 after natural logarithm transformation and the standard deviation (SD) of 0.65 within group on the natural logarithmic scale, with the Cohen’s d value=0.78. Sample sizes were reduced when the feasibility for assays with higher technical requirements were limited, such as PRNT and assays which required greater volumes of blood, such as Omicron-specific tests, as participants with samples tested in earlier timepoints and earlier collection dates or higher blood volume collected chosen. For the proportion of participants with a positive result in immunogenicity outcomes or ARs, with the assumption of a prevalence of 80%, 62 participants would yield a 95% chance to detect the true value within 10% precision.

#### Analysis sets

The primary immunogenicity analysis was performed in healthy participants in the evaluable analysis population. This consisted of participants who were uninfected before and during the study period, which was based on clinical history, baseline S-RBD IgG negativity, and ORF8 IgG negativity, generally healthy status with no major protocol deviations, receipt of dose 3 ≥84 days after dose 1, had blood sampled days 13-42 post-dose 3, and had valid results for the relevant test (Protocol in **Supplementary Materials**). The expanded analysis population were more relaxed, which permitted inclusion of those who received dose 3 ≥56 days after dose 1 and had blood sampled days 6-56 post-dose 3 (Protocol in **Supplementary Materials**). Geometric mean ratios (GMRs) included two-sided 95% CI, corresponding to a one-sided 97.5% CI, which was used for testing non-inferiority at the 0.60 margin. This threshold promotes rapid delivery of study results that requires a smaller sample size amid the evolving pandemic, a practice deemed allowable by the World Health Organization Expert Committee on Biological Standardization and adopted in another recent landmark COVID-19 vaccine study (24, 25). The inferiority analyses were confirmed in the expanded analysis population. Superiority was reached if the lower bound of the 95% CI for GMR was >1, or inferiority was declared if the upper bound of the 95% CI was <1. When both non-inferiority and inferiority were not met, the results were considered as inconclusive. Geometric mean fold rises (GMFR) were calculated for those who had valid results at both timepoints. When there were negative immunogenicity outcome data, values that were half the cut-off were imputed. Unpaired t test after natural logarithmic transformation was performed for comparisons of immunogenicity outcomes between groups. Proportions of positive or negative results were given in percentages with 95% Clopper-Pearson CI. The Fisher exact test was used for comparisons of proportions between groups.

Reactogenicity and safety outcomes were assessed in healthy, uninfected participants who had reported any safety or ARs post-dose 3 and before the study database was locked for this interim analysis in the adolescent group (the healthy safety population). In this primary reactogenicity analysis, the proportions of participants that had reported each of the ARs according to maximum severity and anti-pyretic use were shown as percentages with the 95% Clopper-Pearson CI. The incidences of AEs by severity and SAEs that were reported by the post-dose 3 study visit (28 days after dose 3) were presented as counts and events-per-participant.

#### Vaccine efficacy estimation

Vaccine efficacies (VEs) were estimated as a secondary objective by extrapolation according to the neutralizing titres, as previously established (5, 26). The mean neutralizing level (fold of convalescent) was based on the GMTs of PRNT_50_ for SARS-CoV-2 WT or BA.1 in evaluable adolescents divided by that of 102 convalescent sera from patients aged ≥18 years on days 28-59 after the onset of illness (21, 27). The point estimates of VE were extracted from the best fit of the logistic model using the plot digitizer tool (https://automeris.io/WebPlotDigitizer/, version 4.5).

## Results

### Enrolment and study completion

Among 327 participants in the COVID-19 Vaccination in Adolescents and Children study (COVAC; NCT04800133) who received 2 doses of CoronaVac, 259 participants received a third dose of CoronaVac by January 31, 2022 (**Supplementary Figure 1**). Excluding participants who were infected during the study as determined by ORF8 serology assay or contributed no safety data and did not attend follow-up clinic, 94 adolescents aged 11-17 years and 153 adults aged 18 years or above were included in healthy safety analysis, with comparable demographic characteristics (**Supplementary Table 1**). Doses 1 and 2 were given 28-35 days apart while dose 3 was given at least 84 days after dose 1. Blood sampling was performed on the day of dose 3 and 13-42 days after dose 3. Primary immunogenicity analyses were performed in the evaluable analysis population which included participants with valid and timely immunogenicity results and no protocol deviations (adolescents N=60, adults N=119). Immunogenicity analyses were repeated in the expanded analysis population with relaxed vaccination and blood sampling intervals to further confirm the findings (adolescents N=82, adults N=149; see Methods). Protocol and Statistical Analysis Plan are available in **Supplementary Materials**.

### Immunogenicity outcomes before and after the third dose in adolescents

**Figure 1:**
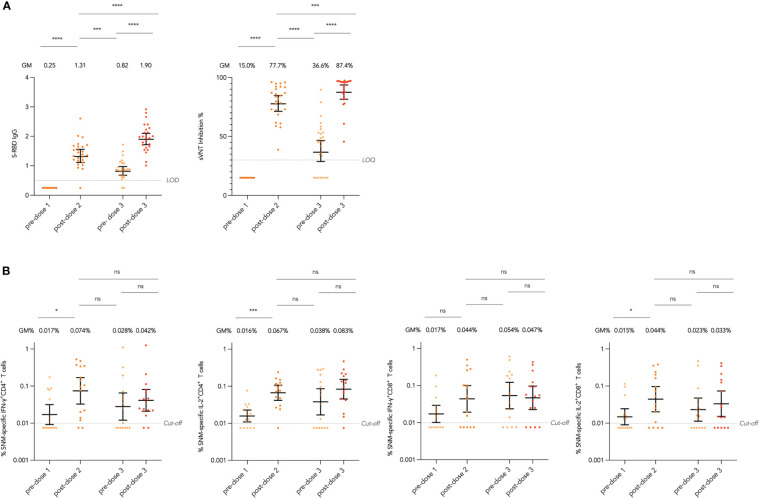
Longitudinal humoral and cellular immunogenicity in healthy evaluable adolescents receiving 3 doses of CoronaVac. **(A)** Longitudinal analysis of Spike receptor-binding domain (S-RBD) IgG OD450 values and surrogate virus neutralisation test (sVNT) inhibition % in evaluable adolescents. **(B)** Longitudinal analysis of total Spike (S), Nucleocapsid (N) and Membrane (M) protein-specific interferon-γ (IFN-γ)+ and interleukin-2 (IL-2)+ CD4+ and CD8+ T cells responses in evaluable adolescents. Geometric means (GM) are shown with centre lines and stated above each column, with corresponding 95% confidence intervals shown by error bars. Samples from the same participant were paired across timepoints and compared with paired t test after natural logarithmic transformation with p-values denoted (*, P<0.05; ***, P<0.001; ****, P<0.0001; ns, not significant). Limits of detection and quantification (LOD and LOQ) and cut-offs were drawn as grey lines.

We first assessed the durability of antibody responses against the WT virus after 2 doses of CoronaVac, including SARS-CoV-2 Spike receptor-binding domain (S-RBD) IgG by enzyme-linked immunosorbent assay (ELISA) and ACE2-blocking antibody by surrogate virus neutralization test (sVNT), as well as interferon-γ (IFN-γ)^+^ and interleukin-2 (IL-2)^+^ CD4^+^ and CD8^+^ T cells responses specific to WT SARS-CoV-2 S, Nucleocapsid (N), and Membrane (M) peptide pools by flow cytometry (see Methods). In evaluable adolescents with paired sera across all timepoints, S-RBD IgG and ACE2-blocking antibody declined significantly with geometric mean (GM) fold reduction of 1.60 and 2.12 fold respectively from post-dose 2 (mean 28 days after dose 2) to pre-dose 3 (**Figure 1A**). Total SNM-specific IFN-γ^+^ and IL-2^+^ CD4^+^ and IL-2^+^ CD8^+^ T cells showed a reducing trend after 2 doses in evaluable adolescents, yet none of the paired analyses between the post-dose 2 and pre-dose 3 timepoints were significant, suggesting T cell responses were preserved (**Figure 1B**). Results for T cell responses to separate S, N and M peptide pools were presented in **Supplementary Figure 2A-C**.

At the post-dose 3 timepoint (mean 19 days after dose 3), evaluable adolescents were assessed for all primary humoral and cellular immunogenicity outcomes against the WT virus (see Methods). All adolescents had positive S-RBD IgG and S-RBD ACE2-blocking antibody post-dose 3 (**Table 1**). On plaque reduction neutralization test (PRNT), 100% and 78.3% adolescents were positive for 50% and 90% PRNT at a limit of detection of 1 in 10, and with GM 50% and 90% PRNT of 55.3 and 17.8 respectively. As CoronaVac is a whole-virion inactivated vaccine, N IgG and N-C terminal domain (N-CTD) IgG were also assessed with 98.3% seropositivity for both. SARS-CoV-2 S IgG, S IgG avidity and S IgG Fcγ receptor IIIa (FcγRIIIa)-binding testing were available in 56 evaluable adolescents, with S IgG and S IgG FcγRIIIa-binding detected in 98.2% tested evaluable adolescents, and GM S IgG avidity of 38.5%. Results in the expanded analysis population were similar (**Supplementary Table 2**). When compared to pre-dose 3 timepoint, evaluable adolescents showed significant GM fold rises in S-RBD IgG of 2.32 fold and sVNT inhibition of 2.39 fold (**Figure 1A**).

**Table 1:** Humoral immunogenicity outcomes against wild-type SARS-CoV-2 after the third dose of CoronaVac in evaluable analysis population.

	Adolescents 3 doses	Adults 3 doses
S IgG on ELISA		
N	56	49
GM OD450 value (95% CI)	0.93 (0.83-1.04)	0.91 (0.83-1.00)
% positive (>/=LOD at 0.3)	98.2%, *P>*0.9999	100%
S-RBD IgG on ELISA		
N	60	119
GM OD450 value (95% CI)	1.77 (1.68-1.87)	1.62 (1.53-1.73)
% positive (>/=LOD at 0.5)	100%, *P>*0.9999	99.2%
S-RBD ACE2-blocking antibody on sVNT		
N	60	119
GM % inhibition (95% CI)	84.9% (81.3-88.6%)	76.9% (71.8-82.3%)
% positive (>/=LOQ at 30%)	100%*, P=*0.30	96.6%
Neutralizing antibody on PRNT		
N	60	22
GM PRNT_90_ (95% CI)	17.8 (13.7-23.3)	12.1 (8.68-16.8)
% positive (>/=LOD at 10)	78.3%, *P=0.57*	72.7%
GM PRNT_50_ (95% CI)	55.3 (43.2-70.7)	32.1 (21.1-48.7)
% positive (>/=LOD at 10)	100%, *P=0.27*	95.5%
S IgG avidity on ELISA		
N	55	49
GM avidity index (95% CI)	38.5% (34.6-42.8)	32.1% (29.4-35.0)
S IgG FcγRIIIa-binding on ELISA		
N	56	49
GM OD450 value (95% CI)	1.41 (1.21-1.63)	1.47 (1.23-1.77)
% positive (>/=LOD at 0.28)	98.2%, *P=*0.60	95.9%
N IgG on ELISA		
N	60	22
GM OD450 value (95% CI)	2.82 (2.62-3.04)	2.08 (1.72-2.52)
% positive (>/=LOD at 0.88)	98.3%*, P=*0.47	95.5%
N-CTD IgG on ELISA		
N	60	22
GM OD450 value (95% CI)	2.97 (2.77-3.17)	1.96 (1.61-2.38)
% positive (>/=LOD at 1.34)	98.3%*, P=*0.17	90.9%

S, spike protein; ELISA, enzyme-linked immunosorbent assay; GM, geometric mean; OD, optical density; LOD, limit of detection; LOQ, limit of quantification; CI, confidence interval; RBD, receptor-binding domain; ACE-2, angiotensin-converting enzyme-2; sVNT, surrogate virus neutralization test; PRNT, plaque reduction neutralization test; PRNT_90_, 90% plaque reduction neutralization titre; PRNT_50_, 50% plaque reduction neutralization titre; FcγRIIIa, Fc gamma receptor III-a; N, nucleocapsid protein; CTD, C-terminal domain. P-values compare the proportion of positive responses between adolescents and adults by Fisher’s exact test.

For cellular immunogenicity outcomes, among 58 evaluable adolescents tested, most participants tested positive for total WT SNM-specific IFN-γ^+^ and IL-2^+^ CD4^+^ T cell responses (74.1% and 79.3% respectively) on flow cytometry at a cut-off of 0.01% (**Table 2**; see Methods). Yet, for IFN-γ^+^ and IL-2^+^ CD8^+^ T cell responses, a lower but still high proportion of participants (62.1% and 65.5%) tested positive. When broken down into T cell responses against separate peptide pools, T cell responses appeared to be lowest for the M peptide pool, which elicited IFN-γ^+^ and IL-2^+^ CD4^+^ T cells in 23.7% and 25.4% tested evaluable adolescents, and IFN-γ^+^ and IL-2^+^ CD8^+^ T cells in 13.6% and 18.6% (**Supplementary Table 3**). Similar results were yielded in the expanded analysis population (**Supplementary Table 4**). When compared to pre-dose 3 timepoint, evaluable adolescents showed statistically insignificant increases in total SNM-specific IFN-γ^+^ and IL-2^+^ CD4^+^ and IL-2^+^ CD8^+^ T cell responses after dose 3 (**Figure 1B**).

**Table 2:** Cellular immunogenicity outcomes against wild-type SARS-CoV-2 after the third dose of CoronaVac in evaluable analysis population.

	Adolescents 3 doses	Adults 3 doses
T cell responses		
Total SNM-specific T cell responses on flow cytometry		
N	58	118
**GM** % IFN-γ^+^CD4^+^ T cells (95% CI)	0.066% (0.041-0.106%)	0.063% (0.045-0.089%)
% positive (>/=cut-off at 0.01%)	74.1%, *P=*0.17	62.7%
**GM** % IL-2^+^CD4^+^ T cells (95% CI)	0.073% (0.049-0.109%)	0.070% (0.052-0.093%)
% positive (>/=cut-off at 0.01%)	79.3%, *P=*0.46	72.9%
**GM** % IFN-γ^+^CD8^+^ T cells (95% CI)	0.071% (0.040-0.125%)	0.051% (0.035-0.075%)
% positive (>/=cut-off at 0.01%)	62.1%, *P=*0.20	50.9%
**GM** % IL-2^+^CD8^+^ T cells (95% CI)	0.041% (0.027-0.063%)	0.034% (0.026-0.044%)
% positive (>/=cut-off at 0.01%)	65.5%, *P=*0.74	61.9%

S, Spike; N, Nucleocapsid; M, Membrane; GM, geometric mean; CI, confidence interval; IFN-γ, interferon-gamma; IL-2, interleukin-2. P-values compare the proportion of positive responses between adolescents and adults by Fisher’s exact test.

### Non-inferiority hypothesis testing of immunogenicity outcomes between adolescents and adults

**Figure 2:**
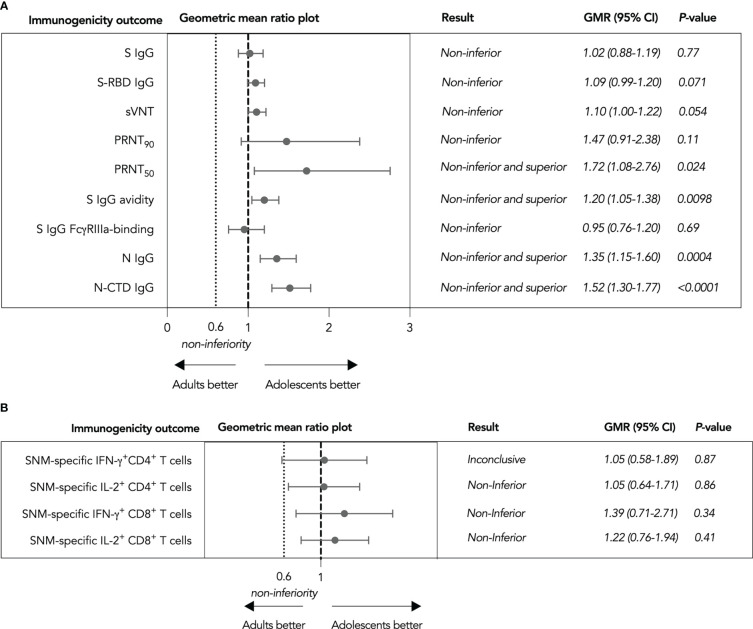
Non-inferiority hypothesis testing of humoral and cellular immunogenicity outcomes against wild-type SARS-CoV-2 after the third dose of CoronaVac in evaluable analysis population. **(A)** Non-inferiority testing of SARS-CoV-2 Spike (S) IgG, S-receptor binding domain (S-RBD) IgG, surrogate virus neutralization test (sVNT), plaque reduction neutralization test (PRNT), S IgG avidity, S IgG Fcγ receptor IIIa (FcγRIIIa)-binding, Nucleocapsid (N) IgG, and N-C-terminal domain (N-CTD) IgG **(B** Non-inferiority testing of total S, N and Membrane (M) protein-specific interferon-γ (IFN-γ)+ and interleukin-2 (IL-2)+ CD4+ and CD8+ T cells Geometric mean ratios (GMR) and two-tailed 95% confidence intervals (CI) were plotted.

To support the use of CoronaVac in adolescents without the availability of efficacy data, we calculated the geometric mean ratios (GMRs) of various immunogenicity outcomes as a primary analysis (see Methods). Nine humoral immunogenicity outcomes assessed were all non-inferior in adolescents as the lower bounds of their two-sided 95% confidence intervals (CI) were at least 0.60 (**Figure 2A**), with 50% PRNT, S IgG avidity, N IgG and N-CTD IgG responses satisfying the criterion for superiority as well. These findings were confirmed by secondary analyses in the expanded analysis population (**Supplementary Figure 3**). On the other hand, total SNM-specific IL-2^+^ CD4^+^ and IFN-γ^+^ and IL-2^+^ CD8^+^ T cells were non-inferior (**Figure 2B**), while it was inconclusive for total SNM-specific IFN-γ^+^ CD4^+^ T cells. When we considered separate S, N and M peptide pools-specific T cell responses, M-specific IL-2^+^ CD4^+^ T cell responses were inferior in evaluable adolescents (**Supplementary Figure 4**). Cellular immunogenicity outcomes were also confirmed in the expanded analysis population, yet total WT SNM-specific IFN-γ^+^ CD4^+^ T cells also tested non-inferior (**Supplementray Figure 5**).

### Humoral and cellular immunogenicity against Omicron in adolescents

**Figure 3:**
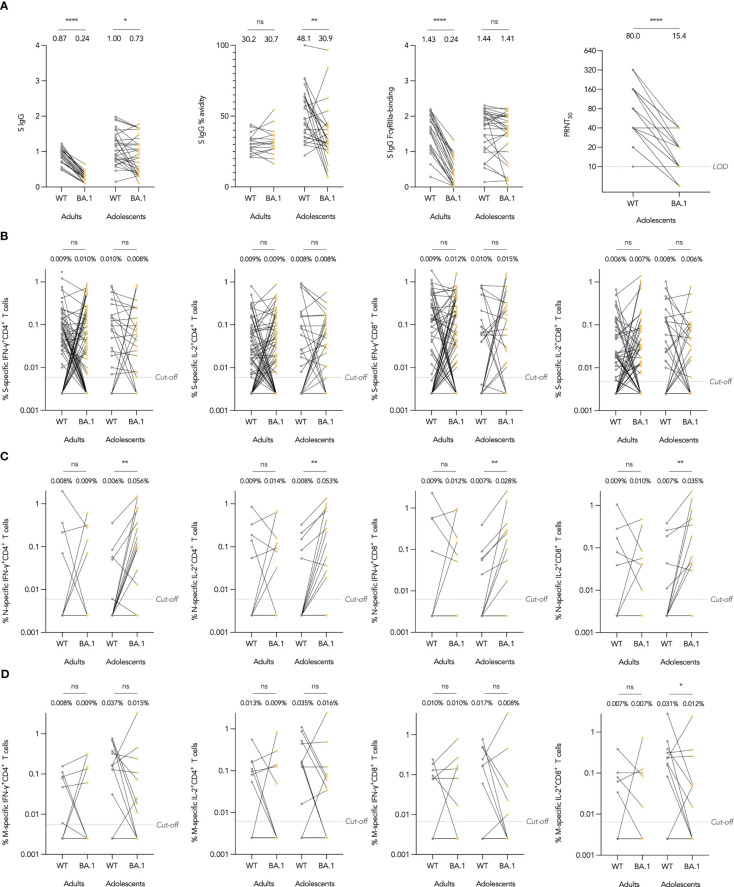
Omicron BA.1-specific humoral and cellular immunogenicity after the third dose of CoronaVac in healthy evaluable adolescents and adults. **(A)** Wild-type (WT) and BA.1 SARS-CoV-2 Spike (S) IgG OD450 values, S IgG avidity index, and S IgG Fcγ receptor IIIa (FcγRIIIa)-binding OD450 values, and 50% plaque reduction neutralization titers (PRNT). **(B-D)** Separate S, N and Membrane (M) protein WT reference pool and BA.1 mutation pool-specific interferon-γ (IFN-γ)+ and interleukin-2 (IL-2)+ CD4+ and CD8+ T cell frequencies. Samples from the same participant were paired between WT and BA.1 and compared with paired t test after natural logarithmic transformation with p-values denoted (*, P<0.05; **, P<0.01; ****, P<0.0001; ns, not significant).

As vaccine efficacy (VE) against SARS-CoV-2 infection may be susceptible to immune escape by novel variants, we included immunogenicity against variants of concern as a secondary objective. At the time of analysis, Omicron has emerged as the dominant variant worldwide and has amino acid substitutions predominantly in the S protein, although also some across the rest of the proteome. We investigated whether Omicron BA.1 could escape S IgG, neutralizing antibodies and T cells elicited by CoronaVac. For Omicron-specific binding antibody responses, we interrogated Omicron BA.1 S IgG binding, avidity, and FcγRIIIa-binding in subsets of adolescents and adults and compared these to the WT assay. As expected, S IgG was significantly reduced in BA.1 compared to WT in adolescents and adults (**Figure 3A**). S IgG avidity was reduced against BA.1 in adolescents as well, yet interestingly, S IgG FcγRIIIa-binding was not significantly reduced. In terms of neutralizing antibodies, GM 50% PRNT was reduced by 5.19 fold against BA.1, but neutralizing antibodies remained detectable in 86.2%.

To assess whether Omicron BA.1 mutations could lead to escape from T cell responses, we focused on BA.1-associated mutations and utilized S, N and M mutation pools which only contained peptides covering BA.1-associated mutations (37, 3 and 3 mutations in S, N and M respectively), and compared their T cell responses against those from WT reference peptide pools containing only the homologous WT peptides (Methods). As expected, no differences between WT and BA.1-S-specific T cells were found in both adolescents and adults (**Figure 3B**). Interestingly, BA.1-associated mutations in N increased IFN-γ^+^ and IL-2^+^ CD4^+^ and CD8^+^ T cell responses, differences which were significant in adolescents (**Figure 3C**). Meanwhile, T cell responses against BA.1 M mutation pool were reduced in comparison to WT reference pool, with the difference significant only for IL-2^+^ CD8^+^ T cells in adolescents, which had a 2.58-fold reduction (**Figure 3D**).

### Reactogenicity and safety of the third dose of CoronaVac in adolescents

**Figure 4:**
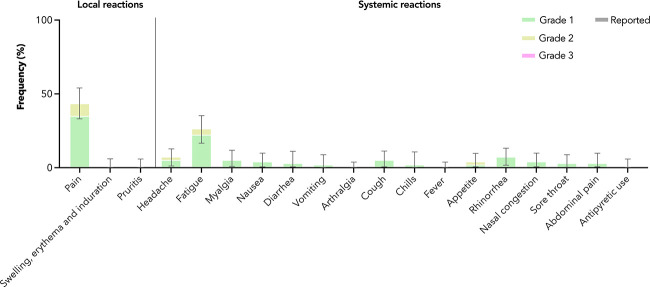
Adverse reactions in adolescents after the third dose of CoronaVac. Adverse reactions were reported by maximal severity (grade 1 – green, grade 2 – yellow, grade 3 – pink) within 7 days after vaccination. Antipyretic use was also captured (reported – grey). 95% confidence intervals are derived from the Clopper-Pearson method and marked by error bars.

Among 94 adolescents in the healthy safety population, very common adverse reactions (ARs) included pain at the injection site (35.1% grade 1 and 8.5% grade 2) and fatigue (22.3% grade 1 and 4.3% grade 2) (**Figure 4**). Almost all ARs reported were of grades 1 and 2 severity; one grade 3 AR (diarrhoea) was reported. Only a single grade 1 adverse event (peripheral swelling) was reported within 28 days after vaccination in adolescents (**Supplementary Table 5**), and it was not considered to have been likely caused by vaccination. There were no serious adverse events reported in the follow-up period.

### Estimation of VE based on neutralization titres against WT and BA.1 SARS-CoV-2 in adolescents

**Figure 5:**
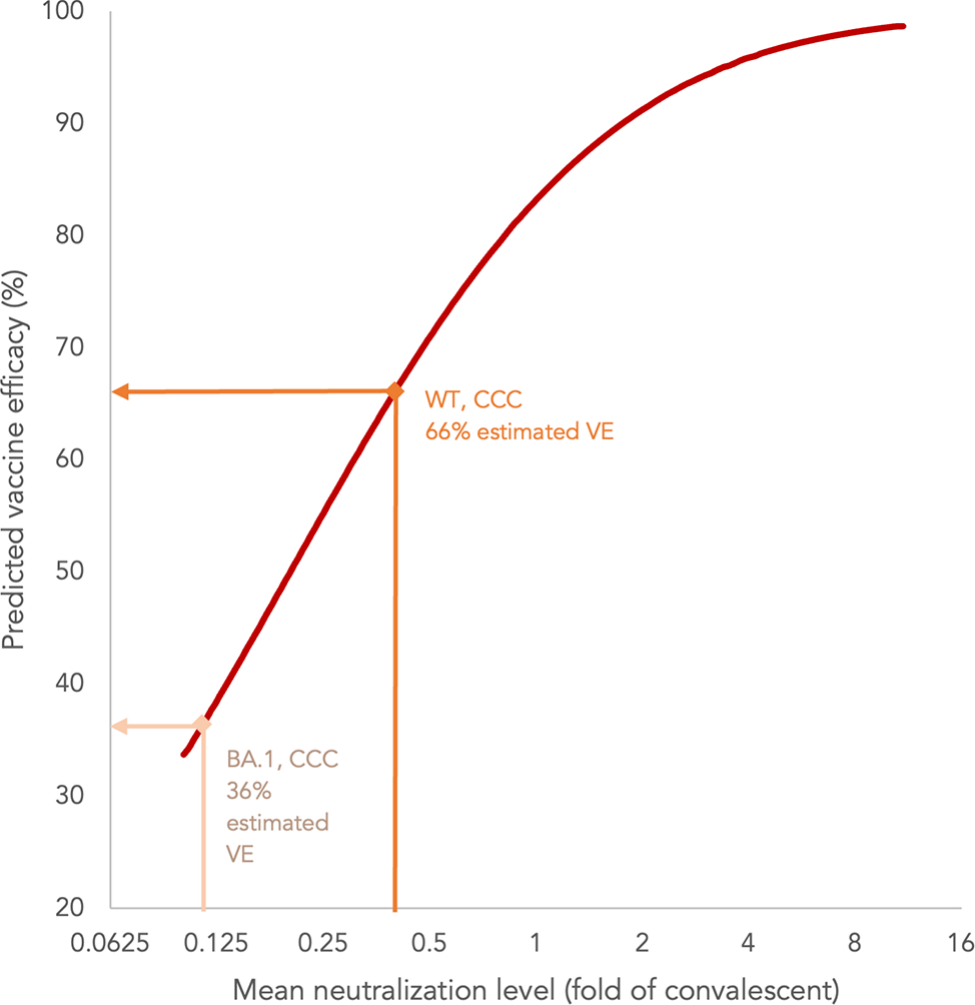
Estimation of vaccine efficacy (VE) of three doses of CoronaVac (CCC) based on neutralization titres against wild-type (WT) and BA.1 SARS-CoV-2 in adolescents.

We extrapolated VE estimates against symptomatic COVID-19 from WT and BA.1 PRNT_50_ results in evaluable adolescents as established by Khoury et al. (Methods) (21, 26, 27). The PRNT results were normalized to 102 in-house convalescent sera collected on days 28-59 post-onset of illness in patients aged ≥18 years, and yielded mean neutralization levels against WT and BA.1 of 0.40 and 0.11, which extrapolated to 66% and 36% VE, respectively (**Figure 5**). These estimates will need to be validated in real-world effectiveness studies.

## Discussion

This study is the first to assess the reactogenicity and immunogenicity of third dose of CoronaVac in healthy adolescents. We found a third dose of CoronaVac further boosted antibody responses after 2 doses in adolescents. Immunobridging analyses showed non-inferior and superior binding and neutralizing antibody responses as well as T cell responses in adolescents when benchmarked against adults. Mutations associated with Omicron BA.1 attenuated binding and neutralizing antibody responses in adolescents who received a third dose, yet binding and neutralizing antibodies remained detectable in most. Adolescents had divergent responses toward mutation pools of Omicron BA.1 S, N and M proteins. ARs were mild, and there were no safety issues observed.

Our finding in adolescents is comparable to that observed in healthy adults who had a further increase in antibody responses after a third dose of CoronaVac (15, 17, 28). As there is evidence of waning protection against symptomatic disease after 2 doses, a booster after the 2-dose primary series of mRNA and adenoviral vector vaccines has been authorized in many countries. For inactivated vaccines, Hong Kong and Singapore have both opined that 3 doses, rather than 2, should form the primary series due to more rapid waning of antibody responses and failure to seroconvert in a minority of healthy vaccinees (29–31). Yet, there have been no published immunogenicity and safety data to inform the use of a third dose in adolescents to date. Our findings of inadequate and rapidly waning antibody responses after 2 doses, and non-inferior antibody responses after a third dose in adolescents compared to adults, support the authorization of a homologous third dose in this age group.

In addition to antibody response, we also surveyed T cell responses, with no waning detected after 2 doses in adolescents, and they were unaltered by a third dose in adolescents. There is limited evidence in literature on waning of T cell responses after 2 doses of CoronaVac in adults. One study in Chile showed an age-dependent preservation of T cell responses with no decline in adults aged 18-59 years and a more significant decline in adults aged 60 years or above (15). In studies of natural infection, half-life of convalescent T cell responses was variably estimated to be 3-7 months (32–35). As our study includes a 3-year follow-up, we will investigate the longevity of T cell responses after a third dose in adolescents. On the other hand, when adolescents were compared against adults after the third dose in our study, T cell responses against SNM in total were non-inferior. Yet, T cell responses against M protein trended lower in adolescents, including IL-2^+^ CD4^+^ T cells which were statistically inferior. Our data hint at differences in targets of T cell reactivity in adolescents versus adults. Previously, our group also found IFN-γ^+^ CD4^+^ and CD8^+^ T cell responses in children aged 1-13 years infected with SARS-CoV-2 appeared to favour non-structural proteins by flow cytometry, although individual proteins were not studied (36). In another study in the United Kingdom where an IFN-γ ELISpot assay was used, T cell responses in seropositive children aged 3-11 years were stronger to the S peptide pool than the combined NM peptide pool, while the responses appeared to be balanced in seropositive adults (37). These observations are possibly due to differential history of antigenic experience with common cold coronaviruses in different age groups, affecting cross-reactive T cell responses (38).

Omicron emerged in most parts of the world during the second year of COVID-19 vaccine rollout, and many studies in adults have pointed to dramatic escape of neutralizing antibodies (8, 9). Sixty-three percent adult vaccinees who received three doses of CoronaVac had detectable neutralizing antibodies against BA.1 in another study by our group (8). Using the same experimental platform, we found sera from a higher proportion (86%) of adolescents who received three doses of CoronaVac neutralized BA.1, suggesting adolescent vaccinees can make more cross-neutralizing antibodies. The neutralization data are in alignment with superior WT S IgG avidity observed in our study, and may lead to preserved VE against symptomatic disease with Omicron. As for T cells, we detected no difference in S-specific T cell response against WT and BA.1 mutated sequences, in agreement with previous studies (10, 11). Interestingly, we found a significant increase in both CD4^+^ and CD8^+^ T cell response against BA.1 mutations in N. It may be because 2 out of 3 mutations in N (31_33delERS, 203_204delRGinsKR) were at the fringes of the immunodominant antigenic regions of WT N protein (39, 40), and the mutations could have enhanced T cell reactivity (41). In contrast, our study revealed CD4^+^ and CD8^+^ T cell responses were both approximately halved against BA.1-associated mutations in M. Divergent changes in T cell responses towards BA.1-associated mutations in different SARS-CoV-2 proteins support that T cells exert very limited or absent selection pressure against SARS-CoV-2 (42). It is also noteworthy while our experimental design allowed us to zoom in on BA.1-associated mutations in each of S, N and M proteins. These changes in T cell response towards mutated sequences, albeit dramatic, should be considered in the context of the entire protein antigen, especially for N and M which contain only three small-scale mutations along the entire protein sequence. Overall, we do not expect a reduction of T cell response or any reduction in vaccine effectiveness against severe disease in vaccinees who received CoronaVac with Omicron BA.1. This conclusion is likely applicable towards other Omicron subvariants, which contain mostly point mutations only, supported by effectiveness data from Hong Kong’s experience with BA.2 (3, 43).

Our study had several strengths and limitations. In addition to neutralizing antibodies, which is a well-established correlate of protection against symptomatic COVID-19 and the basis for other immunobridging studies (26, 44–47), we also studied binding antibodies and T cell responses which also play important roles in protection (48, 49). We were able to track both antibody and T cell responses in healthy adolescent vaccinees from pre-vaccine to post-dose 3, and excluded infection in our participants before or during the study with ORF8 serology at the last timepoint. This was possible also because Hong Kong maintained extremely low levels of local transmission of SARS-CoV-2 during the study period. Non-randomized study design may lead to bias. Sample sizes varied between immunogenicity outcomes as various humoral and cellular assays had different technical and blood volume requirements, and samples were prioritised based on whether the participant had the same test performed at an earlier timepoint, earlier date of sample collection, and sample volume available. We assayed T cell responses by peptide pool-stimulated intracellular IFN-γ and IL-2 cytokine staining, as IFN-γ is an important Th/c1 effector cytokine and IL-2^+^ T cell populations are associated with long-term memory (36, 50, 51). We did not study other antiviral cytokines for polyfunctionality, nor memory and exhaustion markers. We estimated a VE against WT and BA.1 based on PRNT, though that will need to be validated in large-scale effectiveness studies. We only included uninfected adolescents aged 11-17 with good past health in the present analysis, so these findings may not be applicable to infected or younger children as well as paediatric patients with comorbidities. We did not investigate heterologous vaccination or responses against other Omicron subvariants.

In conclusion, our findings support the authorization of a homologous third dose of CoronaVac in healthy adolescents for optimized antibody response. To determine whether a fourth dose of CoronaVac will be needed as a booster in this age group, we will further track the durability of immunogenicity after this third dose and hybrid immunity in this population.

## Data availability statement

The original contributions presented in the study are included in the article/**Supplementary Material**. Further inquiries can be directed to the corresponding authors.

## Ethics statement

The studies involving human participants were reviewed and approved by The University of Hong Kong Institutional Review Board. Written informed consent to participate in this study was provided by the participants, their parents, or legally acceptable representatives.

## Author contributions

YL conceptualized the study. YL, MP, WT, WL, DL, JR, and XW designed the study. YL led the acquisition of funding. YL, WT, and MP supervised the project. SMC, DL, XM, XW, SMSC, IYST, and JHL led the study administrative procedures. WW provided software support. SMC and WW contributed to recruitment of participants. YL and JR provided clinical assessments and follow-up. DL, SMC, JHL, JR, and YL collected safety data. SMSC, SC, KK, KC, JKL, LL, LT, NC, and MP developed and performed S-RBD IgG, N IgG, N-CTD IgG sVNT and neutralization antibody assays. CC and SV developed and performed the S IgG, IgG avidity, S IgG Fcγ receptor IIIa-binding and ORF8 antibody assays. MM provided and developed the specialised ORF8 protein. XW, XM, YZ, MW, WZ, and WT developed and performed the T cell assays. DL and JHL curated and analysed the data. DL, SMSC, YL, and MP performed the vaccine efficacy extrapolation. DL and JHL visualized the data. DL, XM, XW, SMSC, JR, CC, WW, JHL, and SMC validated the data. DL wrote the first draft supervised by YL, with input from JR, XM, XW, SMSC, and CC. All authors contributed to the article and approved the submitted version.
