# Supplementary material for: Immunogenicity against wild-type and Omicron SARS-CoV-2 after a third dose of inactivated COVID-19 vaccine in healthy adolescents
Source: Front Immunol. 2023 Mar 6;14:1106837. doi: 10.3389/fimmu.2023.1106837 (PMC10026957; doi:10.3389/fimmu.2023.1106837)
Supplement: Supplementary file 1 [file DataSheet_1.pdf]

Tables and Figures

Supp. Tables 1-6

Supp. Figs. 1-5

Supp Fig 1 Study completion diagram

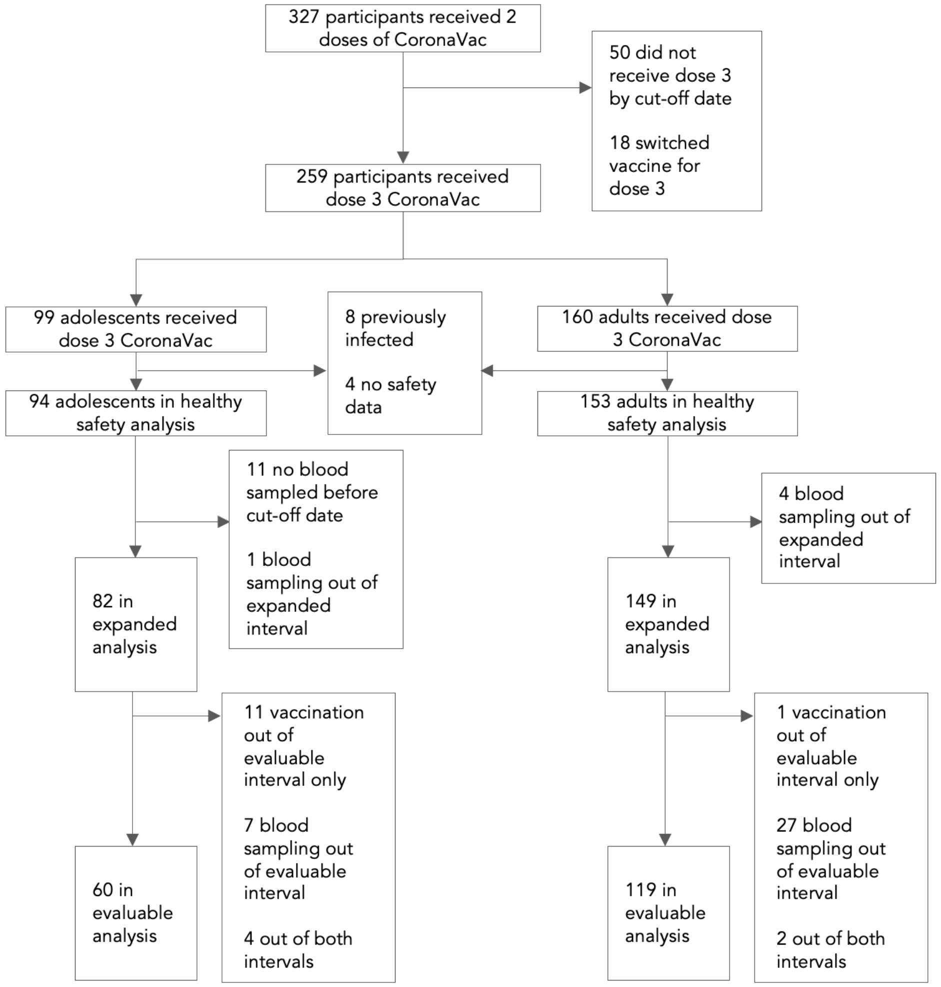

Supp Fig 2 Longitudinal interferon-γ (IFN-γ)⁺ and interleukin-2 (IL-2)⁺ CD4⁺ and CD8⁺ T cells against separate wild-type SARS-CoV-2 Spike (S), Nucleocapsid (N) and Membrane (M) peptide pools in evaluable adolescents receiving 3 doses of CoronaVac

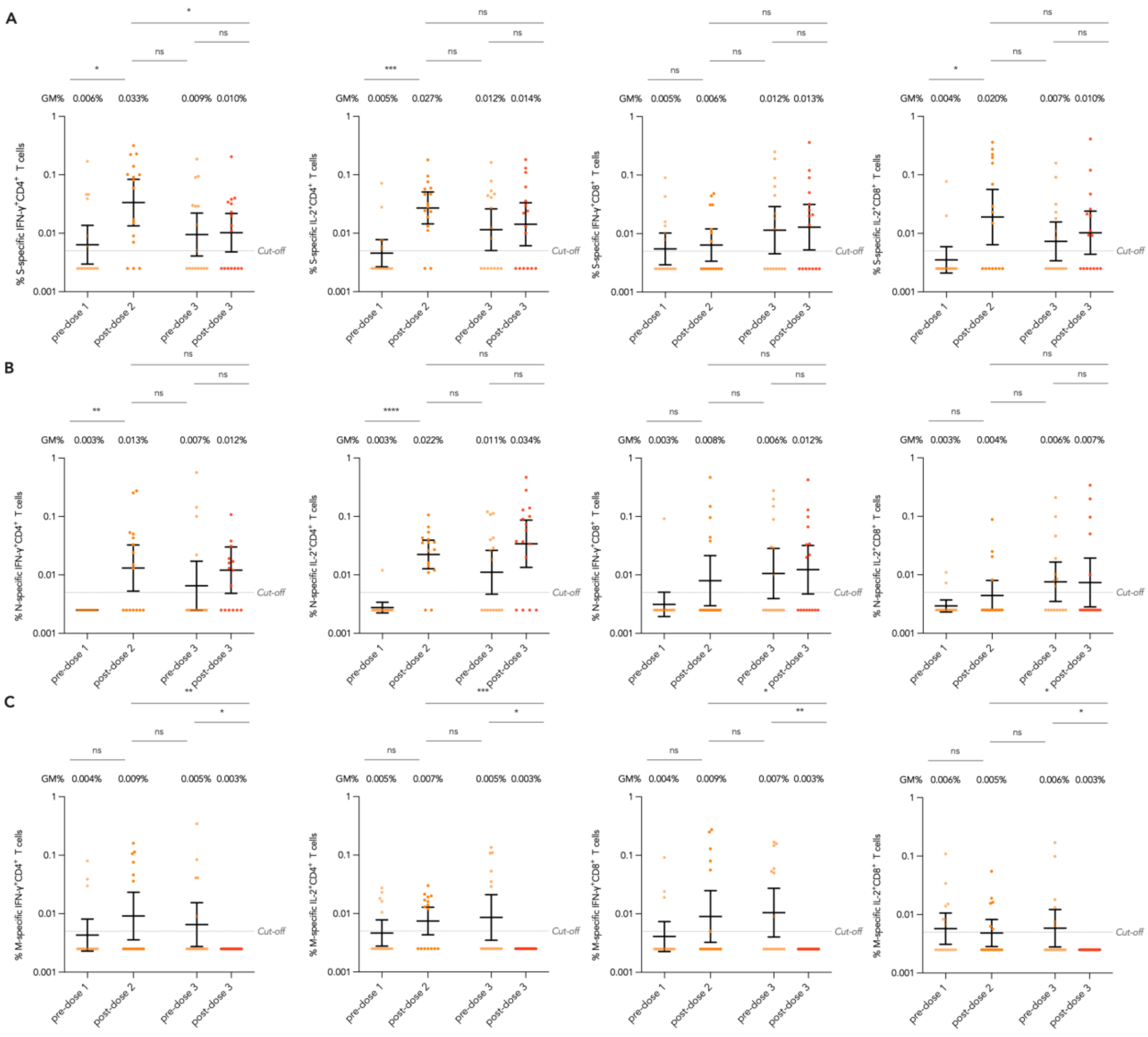

Geometric means (GM) are shown with centre lines and stated above each column, with corresponding 95% confidence intervals shown by error bars. Samples from the same participant were paired across timepoints and compared with paired t test after natural

logarithmic transformation with p-values denoted (*, P<0.05; **, P<0.01; ***, P<0.001; ****, P<0.0001; ns, not significant). Cut-offs are drawn as grey lines.

Supp Fig 3 Non-inferiority hypothesis testing of humoral immunogenicity outcomes against wild-type SARS-CoV-2 after 3 doses of CoronaVac in expanded analysis population

Immunogenicity outcome	Result	GMR (95% CI)	*P*-value
S IgG	*Non-inferior*	*1.03 (0.89-1.20)*	*0.67*
S-RBD IgG	*Non-inferior and superior*	*1.19 (1.06-1.33)*	*0.004*
sVNT	*Non-inferior and superior*	*1.23 (1.10-1.38)*	*0.0004*
$PRNT_{90}$	*Non-inferior*	*1.47 (0.93-2.31)*	*0.097*
$PRNT_{50}$	*Non-inferior and superior*	*1.68 (1.07-2.64)*	*0.026*
S IgG avidity	*Non-inferior and superior*	*1.16 (1.02-1.31)*	*0.021*
S IgG FcγRIIIa-binding	*Non-inferior*	*0.95 (0.77-1.17)*	*0.60*
N IgG	*Non-inferior and superior*	*1.35 (1.11-1.64)*	*0.0026*
N-CTD IgG	*Non-inferior and superior*	*1.48 (1.25-1.74)*	*<0.0001*

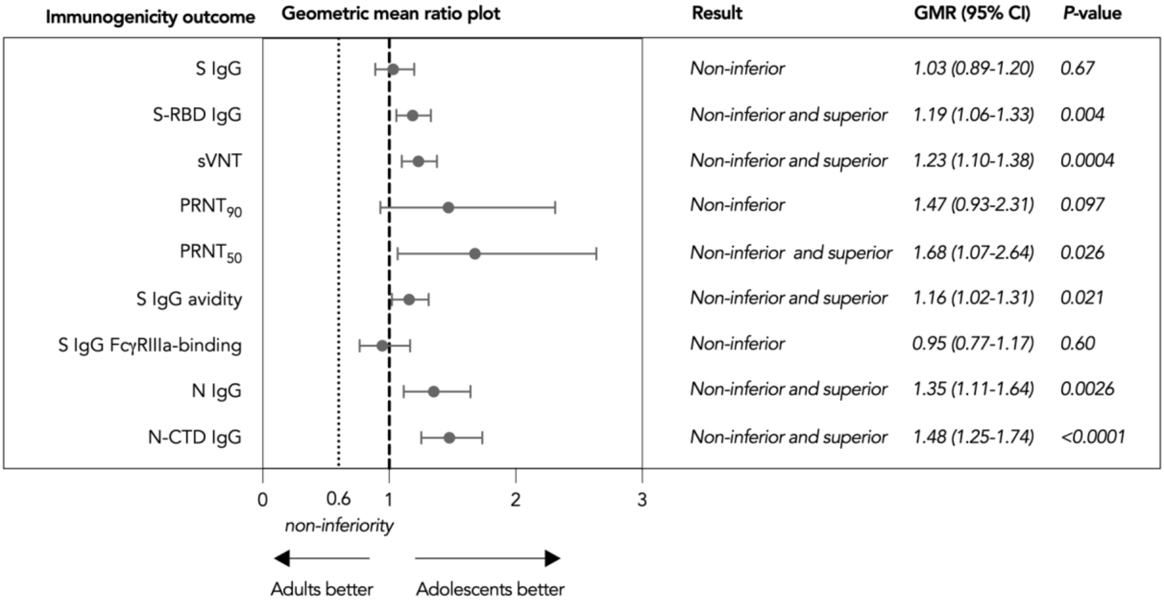

Non-inferiority tests of SARS-CoV-2 Spike (S) IgG, S-receptor binding domain (S-RBD) IgG, surrogate virus neutralization test (sVNT), plaque reduction neutralization test (PRNT), S IgG avidity, S IgG Fcγ receptor IIIa (FcγRIIIa)-binding, Nucleocapsid (N) IgG, and N-C-terminal domain (N-CTD) IgG were performed. Geometric mean ratios (GMR) and two-tailed 95% confidence intervals (CI) were plotted.

Supp Fig 4 Non-inferiority hypothesis testing of separate wild-type SARS-CoV-2 Spike (S), Nucleocapsid (N) and Membrane (M) peptide pools specific cellular immunogenicity outcomes after 3 doses of CoronaVac in evaluable analysis population

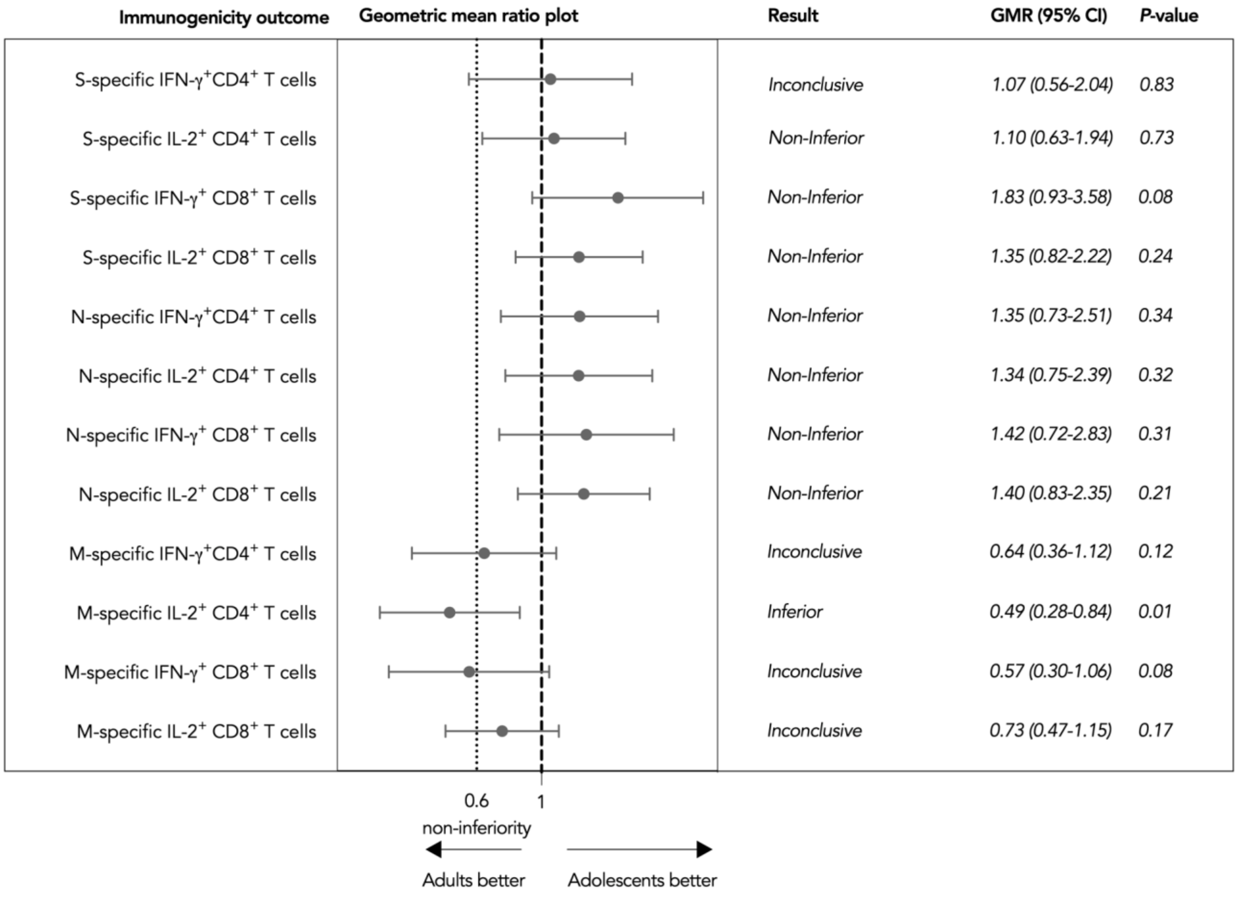

Non-inferiority testing of interferon-γ (IFN-γ)+ and interleukin-2 (IL-2)+ CD4+ and CD8+ T cells specific to separate S, N, and M peptide pools was performed. Geometric mean ratios (GMR) and two-tailed 95% confidence intervals (CI) were plotted.

Supp Fig 5 Non-inferiority hypothesis testing of cellular immunogenicity outcomes after 3 doses of CoronaVac in expanded analysis population

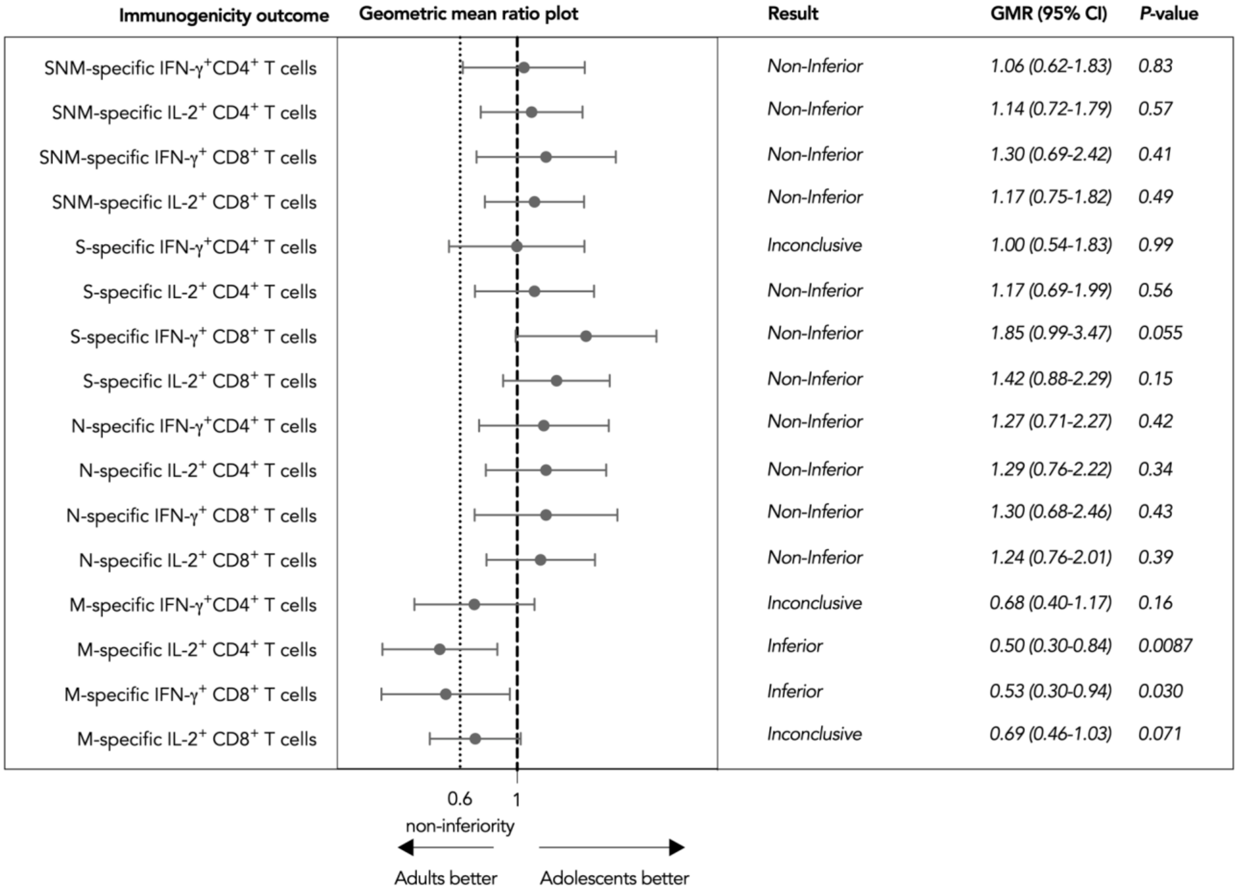

Non-inferiority testing of interferon-γ (IFN-γ)$^{+}$ and interleukin-2 (IL-2)$^{+}$ CD4^{+} and CD8^{+} T cells specific to total and separate Spike (S), Nucleocapsid (N), and Membrane (M) peptide pools was performed. Geometric mean ratios (GMR) and two-tailed 95% confidence intervals (CI) were plotted.

SUPPLEMENTARY TABLES

**Supp Table 1. Participant disposition in the healthy safety population.**	
**Healthy adolescents**	
**Participants**	94
**Male sex**	53 (56.4%)
**Han Chinese**	94 (100%)
**Age (years)**	14.2 (1.75)
**Healthy adults**	
**Participants**	153
**Male sex**	68 (44.4%)
**Han Chinese**	151 (98.7%)
**Age (years)**	48.1 (8.01)
Values are either count (%) or mean (standard deviations).	

Supp Table 2 Wild-type SARS-CoV-2 humoral immunogenicity outcomes after the third dose of CoronaVac in expanded analysis population

	Adolescents<br>3 doses	Adults<br>3 doses
**S IgG on ELISA**		
N	77	60
GM OD450 value (95% CI)	0.87 (0.78-0.97)	0.84 (0.76-0.94)
% positive (>/=LOD at 0.3)	97.4%, *P*>0.9999	98.3%
**S-RBD IgG on ELISA**		
N	82	149
GM OD450 value (95% CI)	1.69 (1.60-1.78)	1.42 (1.31-1.54)
% positive (>/=LOD at 0.5)	100%, *P*=0.16	96.6%
**S-RBD ACE2-blocking antibody on sVNT**		
N	82	149
GM % inhibition (95% CI)	84.0% (81.0-87.1%)	68.3% (62.9-74.1%)
% positive (>/=LOQ at 30%)	100%, *P*=0.0088	92.6%
**Neutralizing antibody on PRNT**		
N	60	25
GM $PRNT_{90}$ (95% CI)	17.8 (13.7-23.3)	12.1 (8.86-16.6)
% positive (>/=LOD at 10)	78.3%, *P=0.58*	72.0%
GM $PRNT_{50}$ (95% CI)	55.3 (43.2-70.7)	32.9 (22.1-49.2)
% positive (>/=LOD at 10)	100%, *P=0.29*	96.0%
**S IgG avidity on ELISA**		
N	75	59
GM avidity index (95% CI)	35.6% (32.5-39.1)	30.8% (28.4-33.4)
**S IgG FcγRIIIa-binding on ELISA**		
N	77	60
GM OD450 value (95% CI)	1.30 (1.14-1.49)	1.38 (1.16-1.64)
% positive (>/=LOD at 0.28)	97.4%, *P*=0.65	95.0%
**N IgG on ELISA**		
N	67	25
GM OD450 value (95% CI)	2.65 (2.42-2.90)	1.96 (1.58-2.43)
% positive (>/=LOD at 0.88)	97.0%, *P*=0.30	92.0%
**N-CTD IgG on ELISA**		
N	67	25
GM OD450 value (95% CI)	2.82 (2.61-3.05)	1.91 (1.61-2.28)
% positive (>/=LOD at 1.34)	97.0%, *P*=0.30	92.0%

S, spike protein; ELISA, enzyme-linked immunosorbent assay; GM, geometric mean; OD, optical density; LOD, limit of detection; LOQ, limit of quantification; CI, confidence interval; RBD, receptor-binding domain; ACE-2, angiotensin-converting enzyme-2; sVNT, surrogate virus neutralization test; PRNT, plaque reduction neutralization test; $PRNT_{90}$, 90% plaque reduction neutralization titre; $PRNT_{50}$, 50% plaque reduction neutralization titre; FcγRIIIa, Fc gamma receptor III-a; N, nucleocapsid protein; CTD, C-terminal domain. *P*-values compare the proportion of positive responses between adolescents and adults by Fisher's exact test.

Supp Table 3 Wild-type SARS-CoV-2 separate S, N and M peptide pools specific cellular immunogenicity outcomes after the third dose of CoronaVac in evaluable analysis population

	**Adolescents 3 doses**	**Adults 3 doses**
**T cell responses**		
**S-specific T cell responses on flow cytometry**		
N	59	118
**GM** % IFN-γ$^+$CD4$^+$ T cells (95% CI)	0.016% (0.010-0.027%)	0.015% (0.010-0.022%)
% positive (>/=cut-off at 0.005%)	57.6%, *P*=0.20	46.6%
**GM** % IL-2$^+$CD4$^+$ T cells (95% CI)	0.017% (0.011-0.027%)	0.015% (0.011-0.021%)
% positive (>/=cut-off at 0.005%)	61.0%, *P*=0.52	55.1%
**GM** % IFN-γ$^+$CD8$^+$ T cells (95% CI)	0.017% (0.010-0.031%)	0.009% (0.006-0.014%)
% positive (>/=cut-off at 0.005%)	49.2%, *P*=0.03	31.4%
**GM** % IL-2$^+$CD8$^+$ T cells (95% CI)	0.009% (0.006-0.015%)	0.007% (0.005-0.009%)
% positive (>/=cut-off at 0.005%)	47.5%, *P*=0.20	37.3%
**N-specific T cell responses on flow cytometry**		
N	58	118
**GM** % IFN-γ$^+$CD4$^+$ T cells (95% CI)	0.014% (0.008-0.024%)	0.010% (0.007-0.015%)
% positive (>/=cut-off at 0.005%)	53.5%, *P=0.11*	39.8%
**GM** % IL-2$^+$CD4$^+$ T cells (95% CI)	0.020% (0.012-0.033%)	0.015% (0.011-0.021%)
% positive (>/=cut-off at 0.005%)	60.3%, *P=0.42*	53.4%
**GM** % IFN-γ$^+$CD8$^+$ T cells (95% CI)	0.015% (0.008-0.028%)	0.010% (0.007-0.015%)
% positive (>/=cut-off at 0.005%)	41.4%, *P*=0.51	35.6%
**GM** % IL-2$^+$CD8$^+$ T cells (95% CI)	0.010% (0.006-0.016%)	0.007% (0.005-0.010%)
% positive (>/=cut-off at 0.005%)	41.4%, *P=0.62*	36.5%
**M-specific T cell responses on flow cytometry**		
N	59	118
**GM** % IFN-γ$^+$CD4$^+$ T cells (95% CI)	0.006% (0.004-0.009%)	0.009% (0.006-0.013%)
% positive (>/=cut-off at 0.005%)	23.7%, *P*=0.12	35.6%
**GM** % IL-2$^+$CD4$^+$ T cells (95% CI)	0.006% (0.004-0.009%)	0.012% (0.009-0.017%)
% positive (>/=cut-off at 0.005%)	25.4%, *P*=0.006	47.5%
**GM** % IFN-γ$^+$CD8$^+$ T cells (95% CI)	0.005% (0.003-0.008%)	0.009% (0.006-0.013%)
% positive (>/=cut-off at 0.005%)	13.6%, *P*=0.02	30.5%
**GM** % IL-2$^+$CD8$^+$ T cells (95% CI)	0.005% (0.003-0.006%)	0.006% (0.005-0.008%)
% positive (>/=cut-off at 0.005%)	18.6%, *P*=0.07	32.2%

S, Spike; N, Nucleocapsid; M, Membrane; GM, geometric mean; CI, confidence interval; IFN-γ, interferon-gamma; IL-2, interleukin-2. *P*-values compare the proportion of positive responses between adolescents and adults by Fisher's exact test.

**Supp Table 4 Wild-type SARS-CoV-2 cellular immunogenicity outcomes after the third dose of CoronaVac in expanded analysis population**		
	**Adolescents 3 doses**	**Adults 3 doses**
**T cell responses**		
**Total S, N, M-specific T cell responses on flow cytometry**		
N	69	122
**GM** % IFN-γ$^+$CD4$^+$ T cells (95% CI)	0.068% (0.045-0.104%)	0.064% (0.046-0.090%)
% positive (>/=cut-off at 0.01%)	73.9%, *P*=0.20	63.9%
**GM** % IL-2$^+$CD4$^+$ T cells (95% CI)	0.079% (0.055-0.112%)	0.069% (0.052-0.091%)
% positive (>/=cut-off at 0.01%)	81.2%, *P=0.29*	73.8%
**GM** % IFN-γ$^+$CD8$^+$ T cells (95% CI)	0.064% (0.038-0.107%)	0.050% (0.034-0.072%)
% positive (>/=cut-off at 0.01%)	59.4%, *P*=0.29	50.8%
**GM** % IL-2$^+$CD8$^+$ T cells (95% CI)	0.039% (0.026-0.057%)	0.033% (0.026-0.043%)
% positive (>/=cut-off at 0.01%)	60.9%, *P>0.9999*	60.7%
**S-specific T cell responses on flow cytometry**		
N	70	122
**GM** % IFN-γ$^+$CD4$^+$ T cells (95% CI)	0.015% (0.010-0.024%)	0.015% (0.011-0.022%)
% positive (>/=cut-off at 0.005%)	54.3%, *P*=0.45	47.5%
**GM** % IL-2$^+$CD4$^+$ T cells (95% CI)	0.018% (0.011-0.027%)	0.015% (0.011-0.021%)
% positive (>/=cut-off at 0.005%)	60.0%, *P*=0.65	55.7%
**GM** % IFN-γ$^+$CD8$^+$ T cells (95% CI)	0.017% (0.010-0.029%)	0.009% (0.006-0.013%)
% positive (>/=cut-off at 0.005%)	48.6%, *P*=0.02	31.2%
**GM** % IL-2$^+$CD8$^+$ T cells (95% CI)	0.010% (0.006-0.015%)	0.007% (0.005-0.009%)
% positive (>/=cut-off at 0.005%)	45.7%, *P*=0.22	36.1%
**N-specific T cell responses on flow cytometry**		
N	69	122
**GM** % IFN-γ$^+$CD4$^+$ T cells (95% CI)	0.013% (0.008-0.021%)	0.010% (0.007-0.015%)
% positive (>/=cut-off at 0.005%)	50.7%, *P*=0.17	40.2%
**GM** % IL-2$^+$CD4$^+$ T cells (95% CI)	0.019% (0.012-0.029%)	0.015% (0.011-0.020%)
% positive (>/=cut-off at 0.005%)	60.9%, *P=0.36*	53.3%
**GM** % IFN-γ$^+$CD8$^+$ T cells (95% CI)	0.013% (0.008-0.023%)	0.010% (0.007-0.015%)
% positive (>/=cut-off at 0.005%)	39.1%, *P*=0.64	35.3%
**GM** % IL-2$^+$CD8$^+$ T cells (95% CI)	0.009% (0.006-0.014%)	0.007% (0.005-0.010%)
% positive (>/=cut-off at 0.005%)	37.7%, *P=0.88*	36.1%
**M-specific T cell responses on flow cytometry**		
N	70	122
**GM** % IFN-γ$^+$CD4$^+$ T cells (95% CI)	0.006% (0.004-0.009%)	0.009% (0.007-0.013%)
% positive (>/=cut-off at 0.005%)	25.7%, *P*=0.15	36.9%
**GM** % IL-2$^+$CD4$^+$ T cells (95% CI)	0.006% (0.004-0.009%)	0.012% (0.009-0.017%)
% positive (>/=cut-off at 0.005%)	27.1%, *P*=0.006	48.4%
**GM** % IFN-γ$^+$CD8$^+$ T cells (95% CI)	0.005% (0.003-0.007%)	0.009% (0.006-0.012%)
% positive (>/=cut-off at 0.005%)	11.4%, *P*=0.004	29.5%
**GM** % IL-2$^+$CD8$^+$ T cells (95% CI)	0.004% (0.003-0.006%)	0.006% (0.005-0.008%)
% positive (>/=cut-off at 0.005%)	15.7%, *P*=0.02	31.2%
S, Spike; N, Nucleocapsid; M, Membrane; GM, geometric mean; CI, confidence interval; IFN-γ, interferon-gamma; IL-2, interleukin-2. *P*-values compare the proportion of positive responses between adolescents and adults by Fisher's exact test.		

**Supp Table 5. Unsolicited adverse events within 28 days of vaccination in adolescent healthy safety population.**	
**Summary of adverse events and severe adverse events**	
**Any adverse event**	1 (0.011)
**Grade 1**	1 (0.011)
**Grade 2**	0 (0.000)
**Grade 3**	0 (0.000)
**Severe**	0 (0.000)
N, total number of participants in adolescent healthy safety population.<br>Data are number of events (events per participant).	

**Supp Table 6. Peptide sequences of SARS-CoV-2 N and M BA.1 and wild-type reference mutation pools**		
	**BA.1 mutation pool**	**Wild-type reference pool**
**SARS-CoV-2 Nucleocapsid protein**		
P13L	GPQNQRNAPRITFGG	GPQNQRNALRITFGG
31_33delERS	GSNQNGERSGARSKQ	GSNQNGGARSKQ
203_204delRGinsKR	STPGSSRGTSPARMA	STPGSSKRTSPARMA
**SARS-CoV-2 Membrane protein**		
D3G	MADSNGTITVEELKK	MAGSNGTITVEELKK
Q19E	LLEQWNLVIGFLFLT	LLEEWNLVIGFLFLT
A63T	WLLWPVTLACFVLAA	WLLWPVTLTCFVLAA
